# Investigations of a Possible Role of SNPs in *KAI1* Gene on Its Down-Regulation in Breast Cancer

**DOI:** 10.31557/APJCP.2020.21.9.2549

**Published:** 2020-09

**Authors:** Haitham Kussaibi, Khaled R. Alkharsah

**Affiliations:** 1 *Department of Pathology, College of Medicine, Imam Abdulrahman Bin Faisal University (IAU), Dammam, Saudi Arabia. *; 2 *Department of Microbiology, College of Medicine, Imam Abdulrahman Bin Faisal University (IAU), Dammam, Saudi Arabia. *

**Keywords:** KAI1 down, regulation, KAI1 gene loss of function, SNPs in KAI1 gene

## Abstract

**Objective:**

*KAI1* (*CD82*) is a metastasis suppressor gene known to be down-regulated in carcinomas of breast, prostate and many other organs. The mechanism of KAI1 down-regulation is complex and not well understood. Here, we investigate the role of 8 SNPs (not previously studied) in *KAI1* gene that could influence its expression in tumor tissue samples of breast cancer patients from the Eastern province of Saudi Arabia.

**Methods:**

Single nucleotide polymorphisms (SNPs) in *KAI1* gene were selected from the NCBI website (dbSNP) and were then filtered for those SNPs causing stop codon mutations (rs139889503 and rs150533529) or nonsynonymous mutation in the 5’-UTR (rs11541048, rs77359459, rs115500759, rs182579675, rs200238062, and rs372733853). SNPs genotyping was performed using TaqMan SNP Genotyping Assay and the results were correlated with KAI1 protein expression profile by immunohistochemistry (IHC) on formalin-fixed paraffin-embedded (FFPE) samples of breast cancer and control none-neoplastic tissues.

**Results:**

*KAI1* expression by IHC was observed in all none-neoplastic breast tissue samples and only in 35% out of the 59 breast cancer tissue samples. None of the samples was homozygous for the stop codon allele A in the SNP rs139889503 or allele T in the SNP rs150533529. The SNPs in the 5-UTR, rs11541048, rs115500759, and rs182579675, were only present in the homozygous state for the G and C alleles respectively in both cancer and control samples. The other SNPs in the 5’-UTR (rs77359459, rs200238062, and rs372733853) had no significant difference in the allele distribution between KAI1 expressing or none-expressing tissue samples.

**Conclusion:**

Our findings showed no significant effect of the studied SNPs on down-regulation of *KAI1* expression.

## Introduction

Breast cancer is a common cancer in the Eastern Province of Saudi Arabia with a high mortality rate which is mostly associated with its dissemination. Mechanism of cancer dissemination, in general, is mediated by complex effects of promoting genes and suppressor genes (Iiizumi et al., 2007). *KAI1 (CD82) *is a well-known metastasis suppressor trans-membrane glycoprotein. KAI1 prevents metastasis by inhibiting cancer cells mobility and infiltration (Liu and Zhang, 2006). To achieve its function, KAI1 protein interacts with other cell membrane proteins such as adhesion proteins, growth factor receptor (GFR), and signaling pathways (Bienstock and Barrett, 2001; Bari et al., 2009). Different types of cancers showed *KAI1* loss of expression especially those in advanced stages (Christgen et al., 2009; Malik et al., 2009a; Malik et al., 2009b; Malik et al., 2009c; Mooez et al., 2011; Shiwu et al., 2012; Scarpino et al., 2013; Zhang et al., 2013). The mechanism behind down-regulation of *KAI1 *expression during cancer development is complex, mediated by transcription control, alternative splicing, and even post-translational remodeling (Tonoli and Barrett, 2005; Tsai et al., 2007; Lee et al., 2011). Only a few studies investigated gene mutation, loss of heterozygosity (LOH), or promoter hypermethylation as a cause of KAI1 down-regulation (Gao et al., 2003; Marreiros et al., 2003). One of the early studies revealed that neither mutation (on 10 prostatic cancer samples) nor allelic loss (on 34 prostatic cancer samples and 12 metastatic lymph nodes) is the cause of *KAI1* gene down-regulation (Dong et al., 1995). A similar observation was made in another study (Miyazaki et al. 2000). Further studies also revealed that epigenetic changes such as methylation of promoter region is not essential to *KAI1* loss of expression (Jackson et al., 2000; Sekita et al., 2001). A later report also showed that loss of *KAI1* expression is not related to mutation, LOH, epigenetic regulation of the promoter, or p53 mutation (Uzawa et al., 2002). On the other hand, a study on 52 ovarian carcinomas found a missense alteration at codon 241 (ATC to GTC), which causes valine to isoleucine substitution in the peptide sequence but it occurs in normal tissues in addition to tumor cells (Liu et al., 2001). One more study discovered a significant correlation between a SNP on exons 3 (-29166 C>T) and the development of colorectal cancer of high grade and advanced stage (Ma et al., 2013).

To our knowledge, after a thorough literature review, no previous study was done on the relationship between the SNPs (selected in our study) and the expression level of KAI1. We aim here to investigate the possibility of a significant association between these selected SNPs, and the *KAI1* expression, which could be if proved, a possible mechanism of *KAI1* loss of expression in breast cancers.

## Materials and Methods

*Sample collection*

Fifty-nine formalin-fixed paraffin-embedded (FFPE) tissue samples of breast cancer in addition to 20 FFPE none-neoplastic breast tissue samples (controls) were collected from the archive of the pathology department at King Fahd Hospital of the University (KFHU). All hematoxylin and eosin slides from tumor and control samples have been examined and verified by a breast pathologist. This study received an ethical approval from the Institutional Review Board (IRB) at Imam Abdulrahman Bin Faisal University (IRB number: IRB–2015-01-095).

*Immunohistochemistry study (IHC)*

Immunohistochemical *KAI1* expression profile of cancer and control samples in the study, has been retrieved from our previous study (Kussaibi et al., 2019) which has been performed on FFPE tumor and control samples’ paraffin sections (3-4μm thick), on Ventana Ultraview Autostainer using Ventana DAB detection universal kit (Ventana, Tucson, AZ, USA) following the standard protocol with further optimization, using as primary antibody (anti-CD82 TS82b, Abcam, Cambridge, UK). The antibody expression level of each sample was classified into: partial, diffuse or no expression.

*DNA extraction*

DNA was extracted from two 10-micrometer tissue sections using the FFPE DNA purification kit (Norgen Biotek, Ontario, Canada) according to the manufacturer’s instructions. The tissue sections were first deparaffinized using 350ul of Qiagen deparaffinization solution for three minutes (Qiagen, Hilden, Germany) followed by 1ml of Xylene for 2 minutes. DNA quality and concentration were assessed using NanoDrop2000 (Life Technologies, California, USA).

*SNP selection*

After screening the *KAI1* gene for SNP using the NCBI website, 3594 SNP were identified in homosapiens *KAI1* gene. All intronic and synonymous SNPs were excluded. The remaining SNPs were selected if they cause stop codon or if they were nonsynonymous and were located in the 5’-untranslated region. The following SNPs were predicted to cause stop or nonsense mutation: rs139889503, rs150533529, rs11541048, rs77359459, rs115500759, rs182579675, rs200238062, rs372733853.

*SNP genotyping*

Genotyping of the KAI1 SNPs was performed by TaqMan assay (Thermo Fisher Scientific, California, USA). The following SNPs were available in the company’s database as validated assays; rs139889503, rs150533529, rs11541048, rs372733853. The other SNPs were ordered as an assay on demand. A twenty-microliter reaction volume containing specific primers and probes for each SNP separately was run on the 7500 Fast real-time PCR thermocycler (ABI, California, USA) according to the manufacturer’s instructions. The software ABI 7500 was used for the endpoint allele calling.

*Data analysis*

Data were collected in an Excel sheet and be prepared for statistical analysis by IBM SPSS software v.24. Pearson’s correlation coefficient (2-tailed) has been performed to measure the strength of the association between each of the mentioned SNPs and the level of *KAI1* expression by IHC. Significant correlation was considered at P **<** 0.050.

**Table 1:** Genotypes of KAI1 SNPs and Their Frequency among KAI1 Expressing and NoneExpressing Tissue Samples in the Study Population

SNP	Genotype	KAI1 expressed	KAI1 Not expressed	*P* -value	Functional Consequence	Validation	MAF
rs139889503	CC	23	35	0.437	stop gained	by cluster	A=0.000016
	AC	1	0				
	AA	0	0				
rs150533529	CC	21	34	0.107	stop gained	by cluster	T=0.000074
	CT	3	1				
	TT	0	0				
rs11541048	GG	24	35	-	5’-UTR variant	by cluster	A=0.00003
	AA/AG	0	0				
rs77359459	GG	22	32	0.974	5’-UTR variant	by 1000G by cluster by frequency	C=0.0308
	GA	2	3				
	AA	0	0				
rs115500759	CC CT/TT	24 0	35 0	0.437	5’-UTR variant	by 1000G by cluster by frequency	T=0.0056
rs182579675	CC	24	35	-	5’-UTR variant	by 1000G	T=0.0002
	CT/TT	0	0				
rs200238062	GG	22	34	0.638	5’-UTR variant	by 1000G	T=0.0002
	GA	2	1		by cluster		
	AA	0	0				
rs372733853	TT	24	34	0.453	5’-UTR variant	no info	no info
	TC	0	1				
	CC	0	0				

## Results

*The expression of KAI1 by IHC*

In a previous study, we investigated the expression level of *KAI1* by immunohistochemistry from breast cancer tissue and none-neoplastic breast tissues using anti-CD82 TS82B (Kussaibi et al., 2019). Out of 59 tumor samples included in the current study, 35% (n=22/59) showed expression of KAI1 protein (partial or diffuse). The remaining 65% (n=37/59) showed loss of *KAI1 *expression. All the 20 control samples showed expression of *KAI1*.

*Genotyping of the selected SNPs*

We searched the dbSNP database for SNPs in the gene coding region for *KAI1*. Over three thousand SNPs were identified in the homosapiens *KAI1* gene. We have then excluded all SNPs that are intronic or produce synonymous mutations. The remaining SNPs were further refined to SNPs which produce a stop codon (rs139889503 and rs150533529) or if they were nonsynonymous mutations and were in the 5’-untranslated region (Table 1). None of the patients or controls in the study was homozygous for the stop-coding nucleotide (A) in the SNP rs139889503. Only one tumor patient was heterozygous for this SNP and the expression of *KAI1* was not affected. Similarly, none of the patients or controls in the study was homozygous for the stop-coding nucleotide (T) in the second SNP rs150533529. However, 4 cases, including two normal controls, were heterozygous for this SNP (Table 1). The normal tissue showed expression of *KAI1* in the two cases. While in the tumor samples, KAI1 was expressed in 50% (n=1/2) of the cases. 

We also have looked for SNPs causing non-synonymous mutations in the 5’-UTR of KAI1 expecting them to affect *KAI1* expression and identified six non-synonymous SNPs (Table 1). All the patient’s samples and control samples were homozygous for the SNP rs182579675. Other SNPs were also not found in the homozygous state for the minor allele and there was no significant effect on the expression of *KAI1* in the heterozygous state. Most of tumor and control samples showed almost the same SNP profile with no significant association with *KAI1* expression status (Table 1). 

## Discussion

KAI1 is a transmembrane glycoprotein that inhibits tumor metastasis by inducing senescence of tumor migrating cells at the endothelial surface through interaction with Duffy antigen chemokine receptor (Bandyopadhyay et al., 2006). Therefore, its expression is usually downregulated in many tumors particularly in advance stages. Understanding the mechanism of *KAI1* loss of expression in advanced tumor is crucial to understand the mechanism of metastasis inhibition. A polymorphism in the *KAI1* gene or its regulatory region that may accumulate during the tumor growth could affect its expression and lead to reduced translation or complete loss of expression.

Our findings showed that most of tumor and control samples in the study showed almost the same SNP profile with no significant association with *KAI1* expression status in breast cancer tissues. We investigated the effect of SNPs that may produce stop codon and lead to truncation of the protein on *KAI1* expression. None of the samples was homozygous for the allele that produces stop mutation. However, four cases, including two normal controls, were heterozygous for these SNPs. The normal tissue showed expression of* KAI1* in the two cases. While in the tumor samples, KAI1 was expressed in 50% (n=1/2) of the cases. This indicates that the heterozygous state of these two SNPs does not affect* KAI1* expression.

Similarly, no effect was observed for the non-synonymous SNPs in the 5’-untranslated region (5’-UTR) of the *KAI1* gene on its expression. The 5’-UTR is an untranslated sequence in an mRNA directly upstream of the start codon, which plays an important role in the regulation of protein expression by influencing the translation of its transcript. 

These SNPs were not previously studied, and no data was available about them in the literature other than the databases. A study investigating SNP in multiple metastasis inhibitor genes including KAI1 did not find a significant association between particular SNP and risk of breast cancer but rather found an effect of multiple variants in several genes including *KAI1* on the risk of breast cancer when there is lymph nodes involvement (Roberts et al., 2017). Therefore, there could be no one SNPs that by itself affects the* KAI1* expression but rather a combined effect of multiple SNPs.

A study on patients with head and neck cancer concluded that germline mutation in *KAI1* gene could be a less frequent event (Nazir et al., 2011). 

The small sample size could be a limitation in our study. Therefore, further studies are required to understand KAI1 down-regulation in tumor tissue which could be not only at the germline level but also at the transcriptional or translational level (epigenetic regulation) (Lee et al., 2017).

In conclusion, our findings did not reveal significant association between the studied SNPs and the *KAI1* expression in breast cancer, which again makes mutations a less likely mechanism of KAI1 loss of expression. 

## Data Availability

The data file related to this study is available from the corresponding author on reasonable request.
